# Supplementary material for: Extensive T-Cell Profiling Following SARS-CoV-2 mRNA Vaccination in Multiple Sclerosis Patients Treated with DMTs
Source: Pathogens. 2025 Feb 27;14(3):235. doi: 10.3390/pathogens14030235 (PMC11944680; doi:10.3390/pathogens14030235)
Supplement: Supplementary file 1 [file pathogens-14-00235-s001.zip › pathogens-3491558-supplementary.pdf]

Supplementary Material

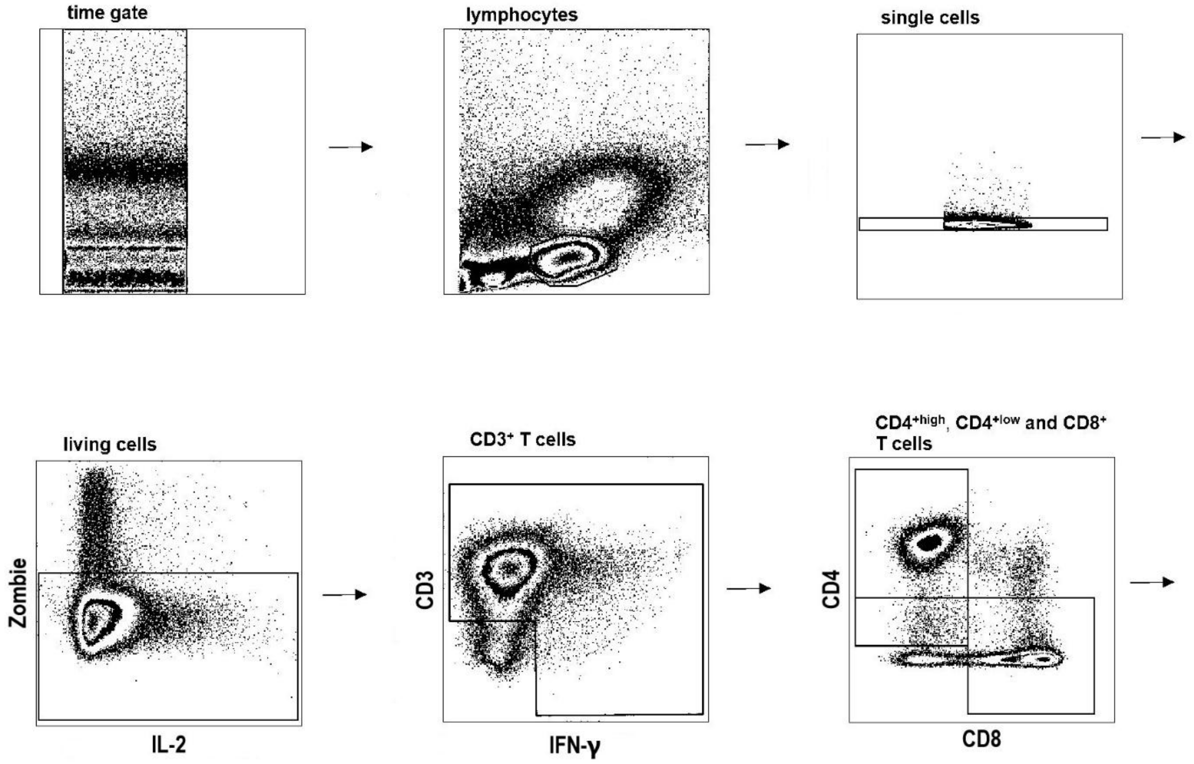

surface activation markers on CD4⁺ (A) and CD8⁺ (B) T cells

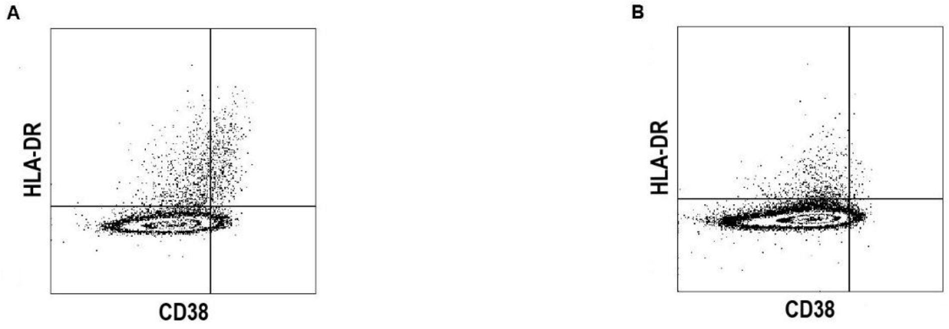

surface differentiation markers on CD4⁺ (C) and CD8⁺ (D) T cells

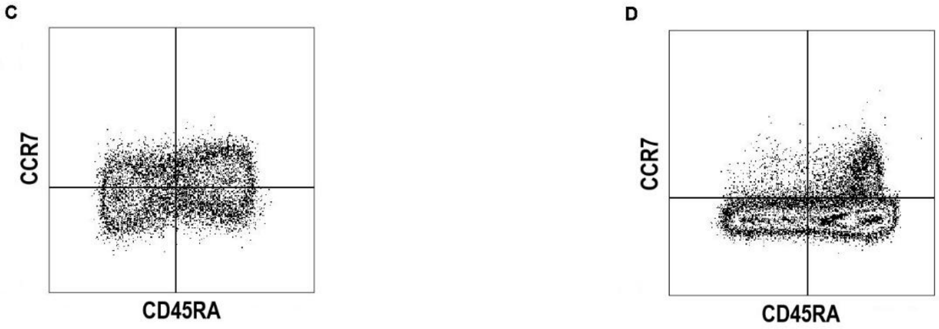

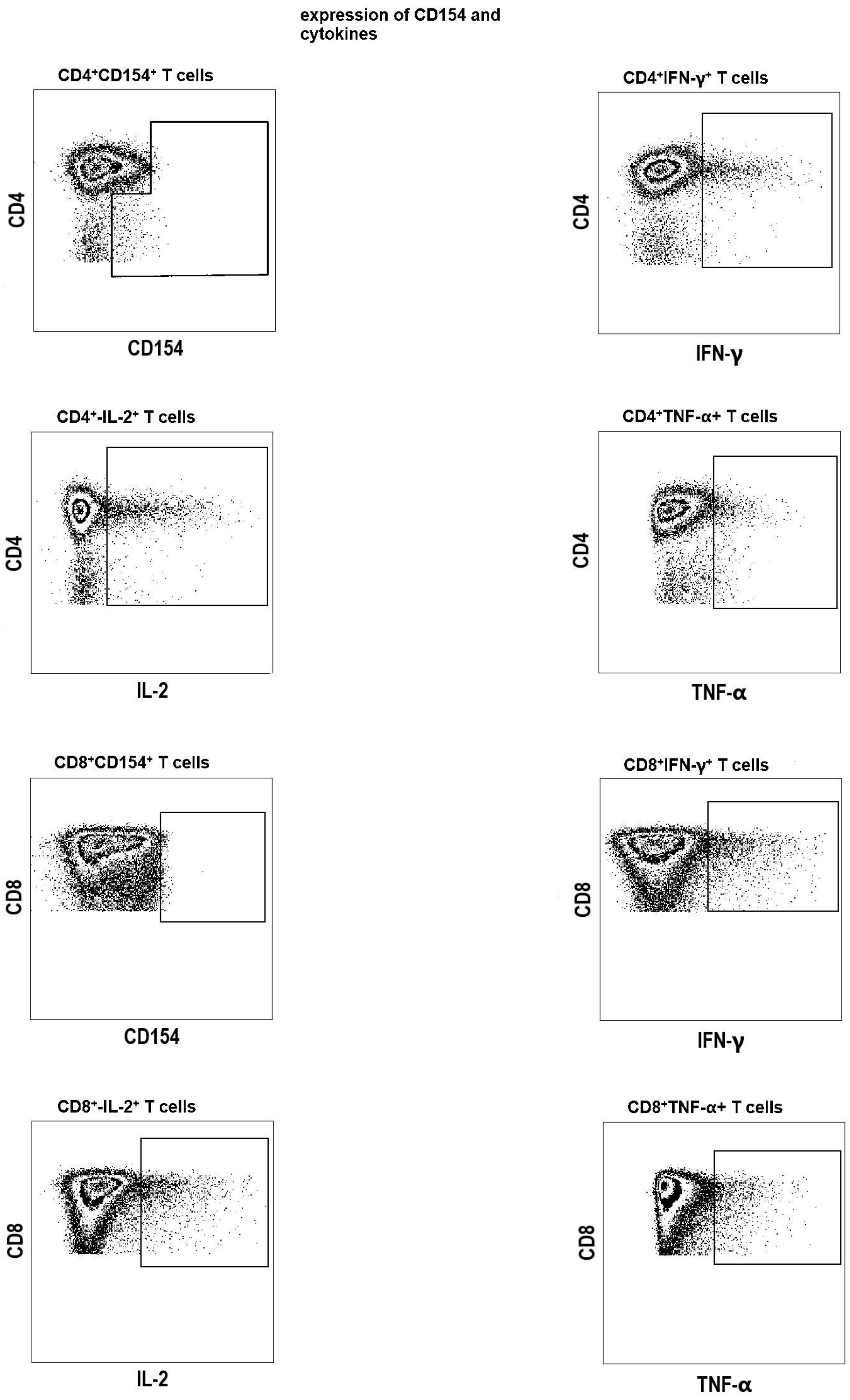

Figure S1. Gating strategy for characterization of SARS-CoV-2 specific T cells. Shown are the gating steps used to differentiate cell populations in relative percentages of $CD4^+$ and $CD8^+$ T cells as well as their subpopulations, including surface activation markers, surface differentiation markers, CD154 and cytokines.

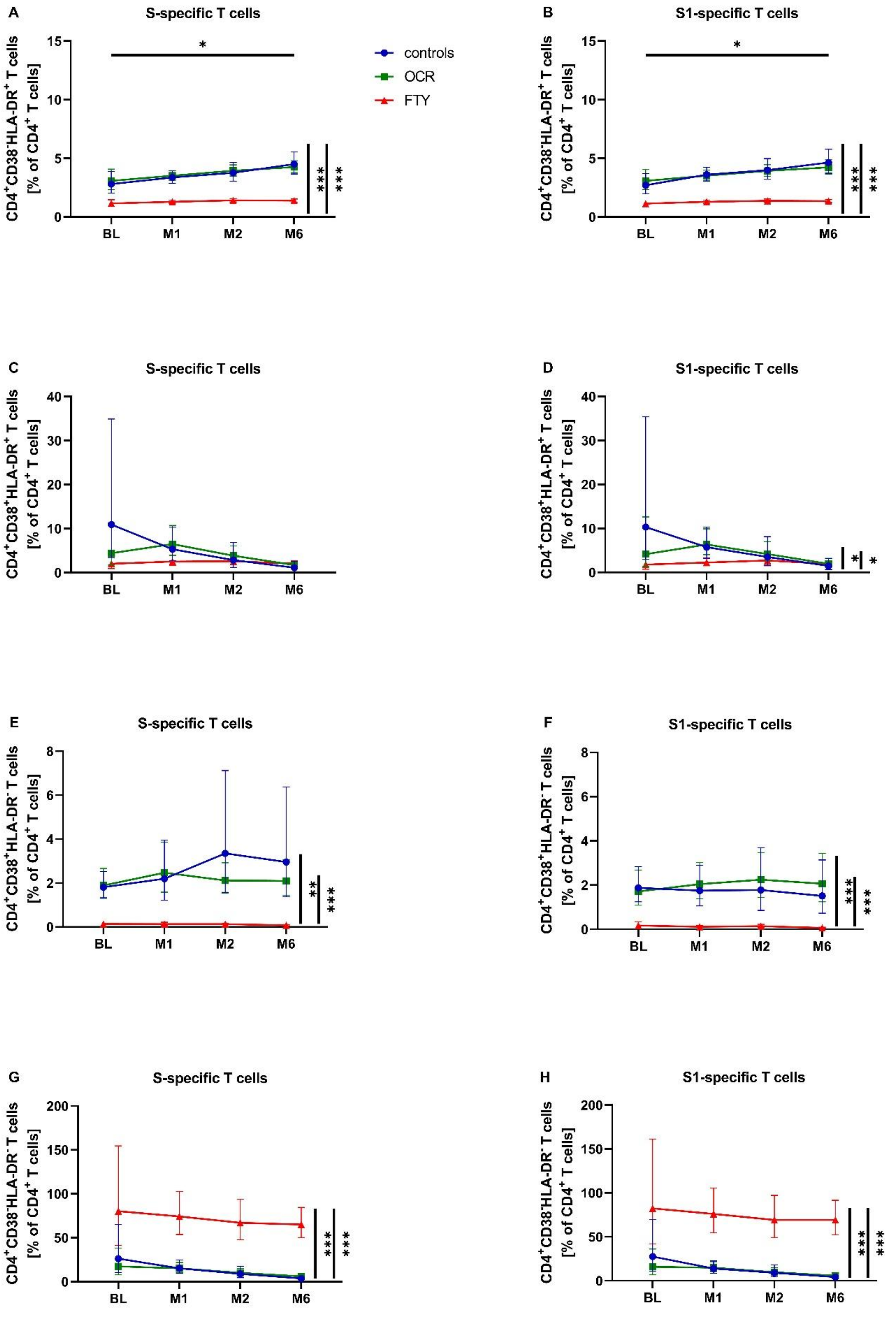

Figure S2. Impact of first SARS-CoV-2 mRNA vaccination on selected spike-specific (S) T-cell subsets and T-cell subsets specific for the S1 domain of the spike protein (S1) in the control group (controls, $n = 17$) compared to the fingolimod (FTY, $n = 15$) and the ocrelizumab (OCR, $n = 15$) group. Relative percentages of T-cell subsets are shown at baseline (BL), one-month follow-up (M1), two-month follow-up (M2) and six-month follow-up (M6). Means with 95% confidence intervals are presented for relative percentages of CD4$^+$CD38$^-$HLA-DR$^+$ T cells **(A,B)**, CD4$^+$CD38$^+$HLA-DR$^+$ T cells **(C,D)**, CD4$^+$CD38$^+$HLA-DR$^-$ T cells **(E,F)** and CD4$^+$CD38$^-$HLA-DR$^-$ T cells **(G,H)**. Mirror-transformed (reflected) data: CD4$^+$CD38$^-$HLA-DR$^+$ T cells, CD4$^+$CD38$^-$HLA-DR$^-$ T cells. Asterisks indicate a statistically significant difference in percentages of relative T-cell subsets between selected time points or groups (* $p < 0.05$, ** $p < 0.01$ *** $p < 0.001$).

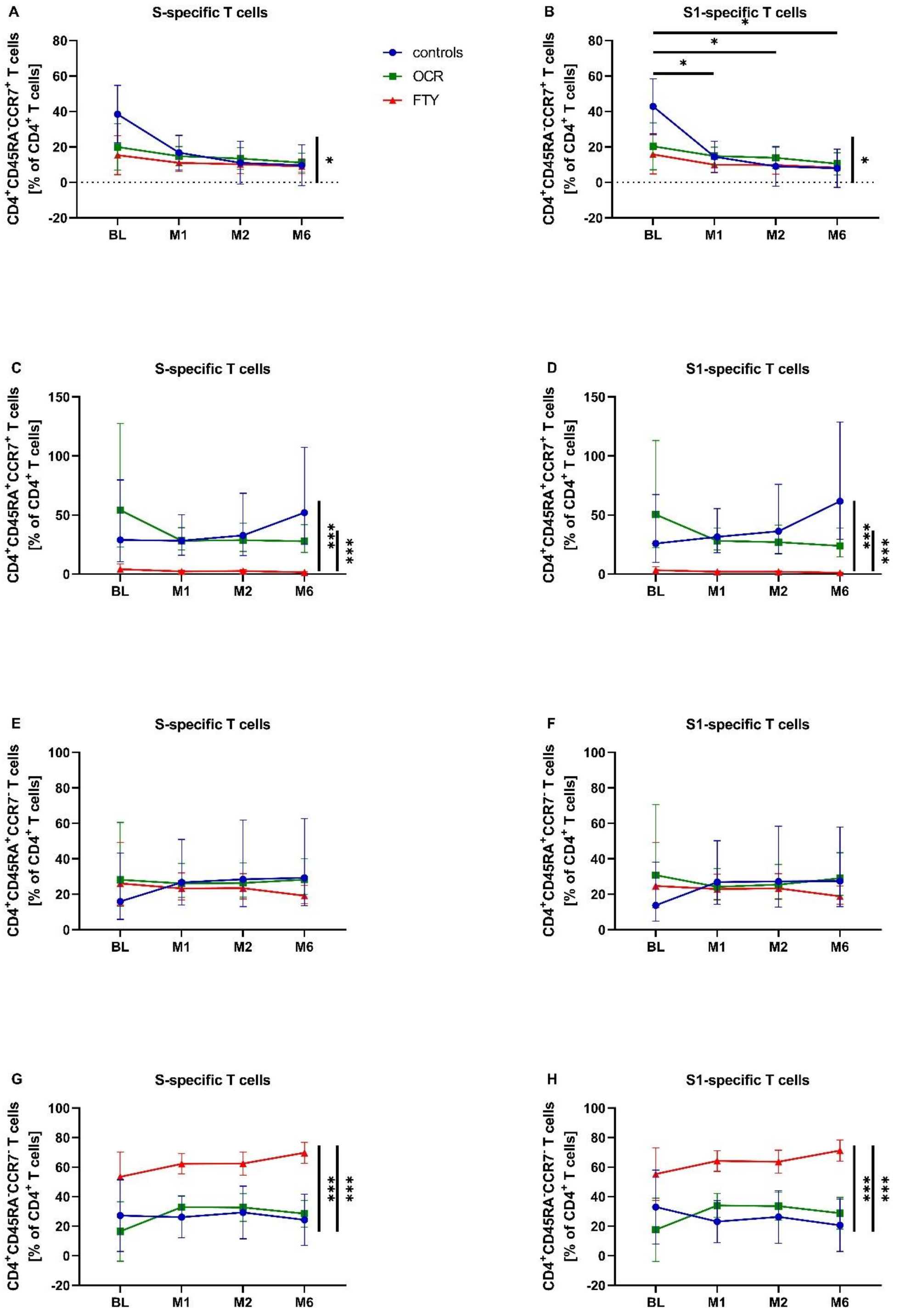

Figure S3. Impact of first SARS-CoV-2 mRNA vaccination on selected spike-specific (S) T-cell subsets and T-cell subsets specific for the S1 domain of the spike protein (S1) in the control group (controls, $n = 17$) compared to the fingolimod (FTY, $n = 15$) and the ocrelizumab (OCR, $n = 15$) group. Relative percentages of T-cell subsets are shown at baseline (BL), one-month follow-up (M1), two-month follow-up (M2) and six-month follow-up (M6). Means with 95% confidence intervals are presented for relative percentages of $CD4^{+}CD45RA^{-}CCR7^{+}$ T cells **(A,B)**, $CD4^{+}CD45RA^{+}CCR7^{+}$ T cells **(C,D)**, $CD4^{+}CD45RA^{+}CCR7^{-}$ T cells (**E**,**F**) and $CD4^{+}CD45RA^{-}CCR7^{-}$ T cells **(G,H)**. Asterisks indicate a statistically significant difference in percentages of relative T-cell subsets between selected time points or groups (* $p < 0.05$, *** $p < 0.001$).

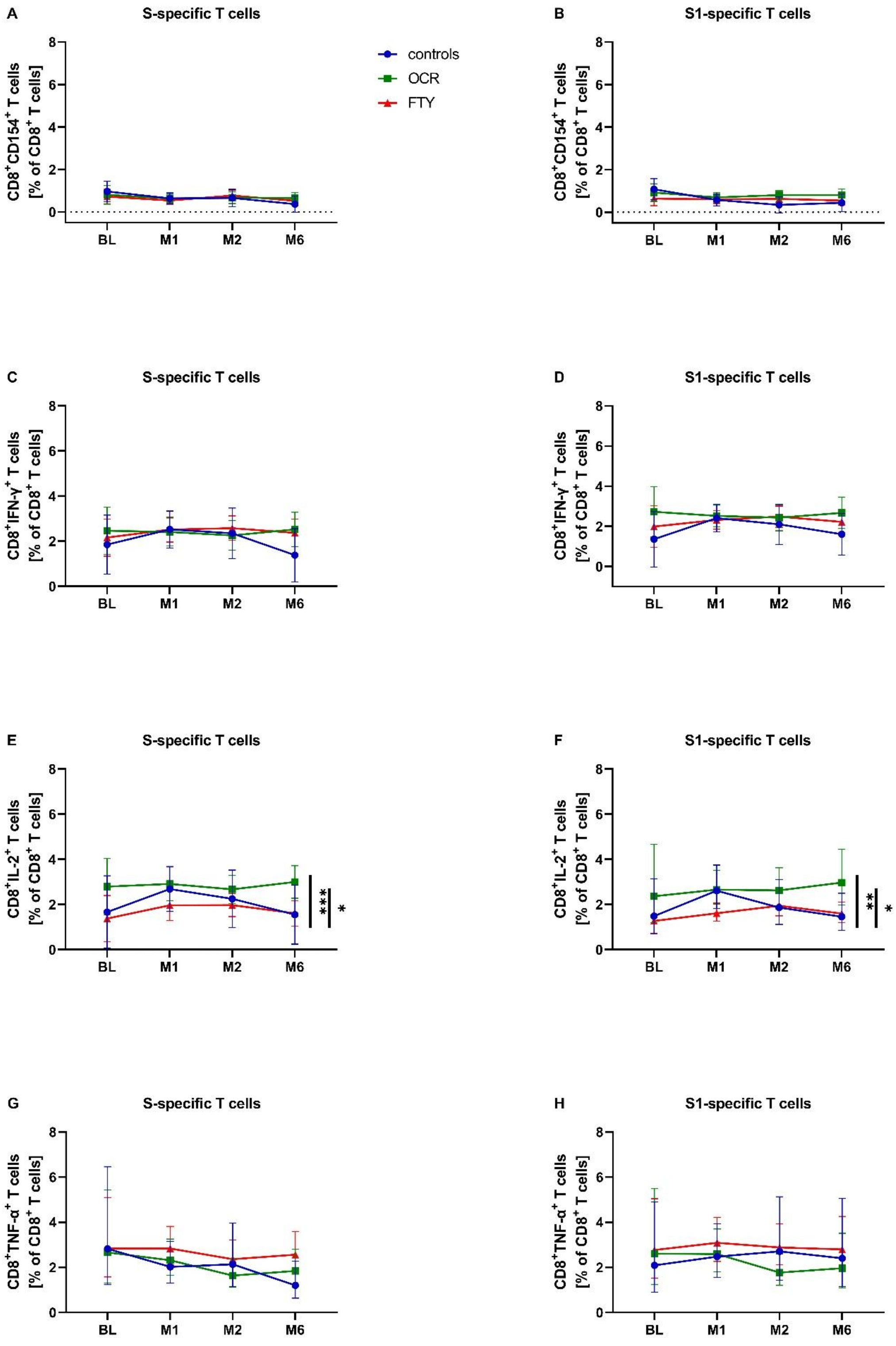

Figure S4. Impact of first SARS-CoV-2 mRNA vaccination on selected spike-specific (S) T-cell subsets and T-cell subsets specific for the S1 domain of the spike protein (S1) in the control group (controls, $n = 17$) compared to the fingolimod (FTY, $n = 15$) and the ocrelizumab (OCR, $n = 15$) group. Relative percentages of T-cell subsets are shown at baseline (BL), one-month follow-up(M1), two-month follow-up (M2) and six-month follow-up (M6). Means with 95% confidence intervals are presented for relative percentages of $CD8^{+}CD154^{+}$ T cells **(A,B)**, $CD8^{+}IFN\text{-}\gamma^{+}$ T cells **(C,D)**, $CD8^{+}IL\text{-}2^{+}$ T cells (**E,F**) and $CD8^{+}TNF\text{-}\alpha^{+}$ T cells (**G,H**). Asterisks indicate a statistically significant difference in percentages of relative T-cell subsets between selected time points or groups (* $p < 0.05$, ** $p < 0.01$, *** $p < 0.001$).

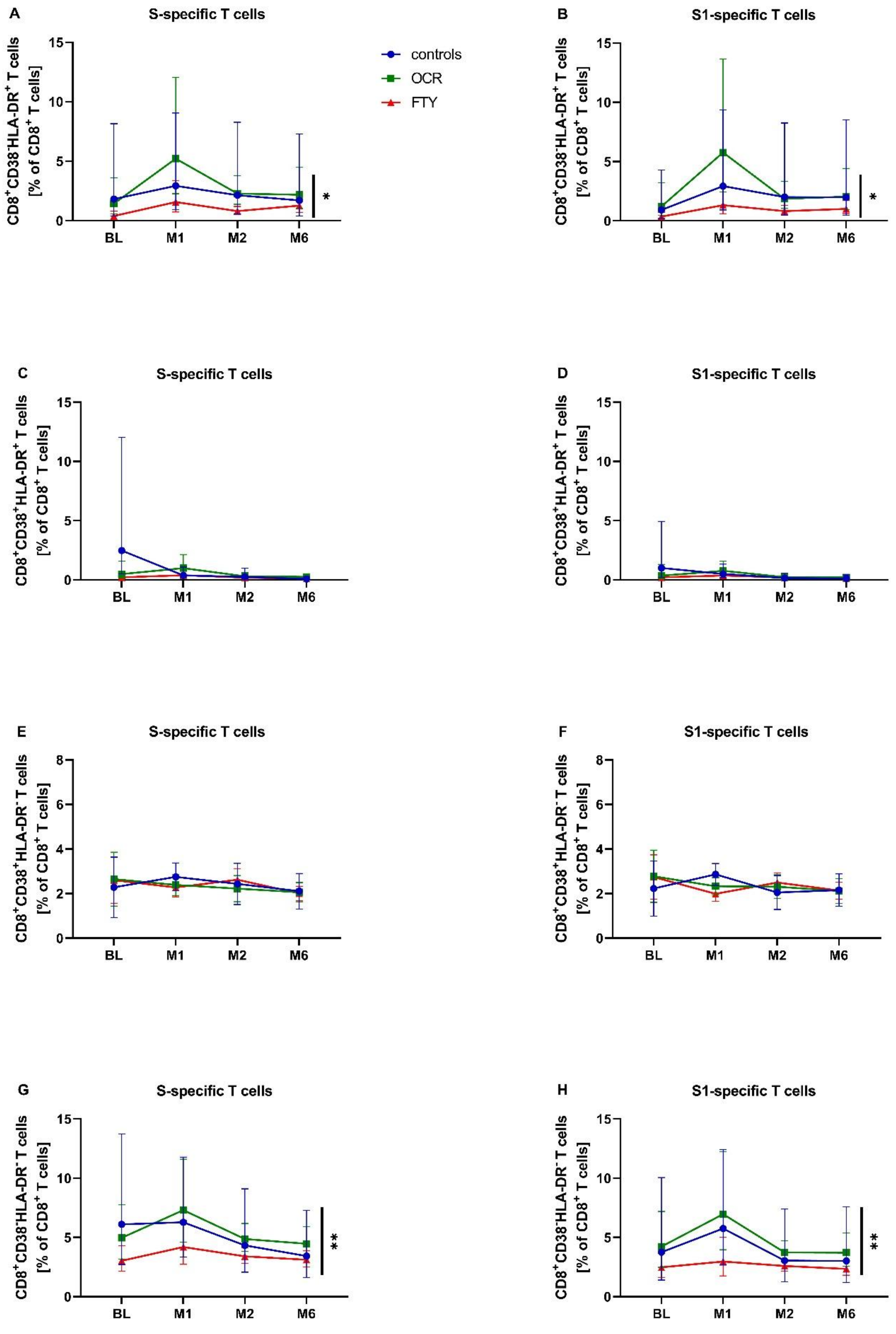

Figure S5. Impact of first SARS-CoV-2 mRNA vaccination on selected spike-specific (S) T-cell subsets and T-cell subsets specific for the S1 domain of the spike protein (S1) in the control group (controls, $n = 17$) compared to the fingolimod (FTY, $n = 15$) and the ocrelizumab (OCR, $n = 15$) group. Relative percentages of T-cell subsets are shown at baseline (BL), one-month follow-up (M1), two-month follow-up (M2) and six-month follow-up (M6). Means with 95% confidence intervals are presented for relative percentages of CD8$^+$CD38$^-$HLA-DR$^+$ T cells **(A,B)**, CD8$^+$CD38$^+$HLA-DR$^+$ T cells **(C,D)**, CD8$^+$CD38$^+$HLA-DR$^-$ cells **(E,F)** and CD8$^+$CD38$^-$HLA-DR$^-$ T cells **(G,H)**. Mirror-transformed (reflected) data: CD8$^+$CD38$^-$HLA-DR$^-$ T cells. Asterisks indicate a statistically significant difference in percentages of relative T-cell subsets between selected time points or groups (* $p < 0.05$, ** $p < 0.01$).

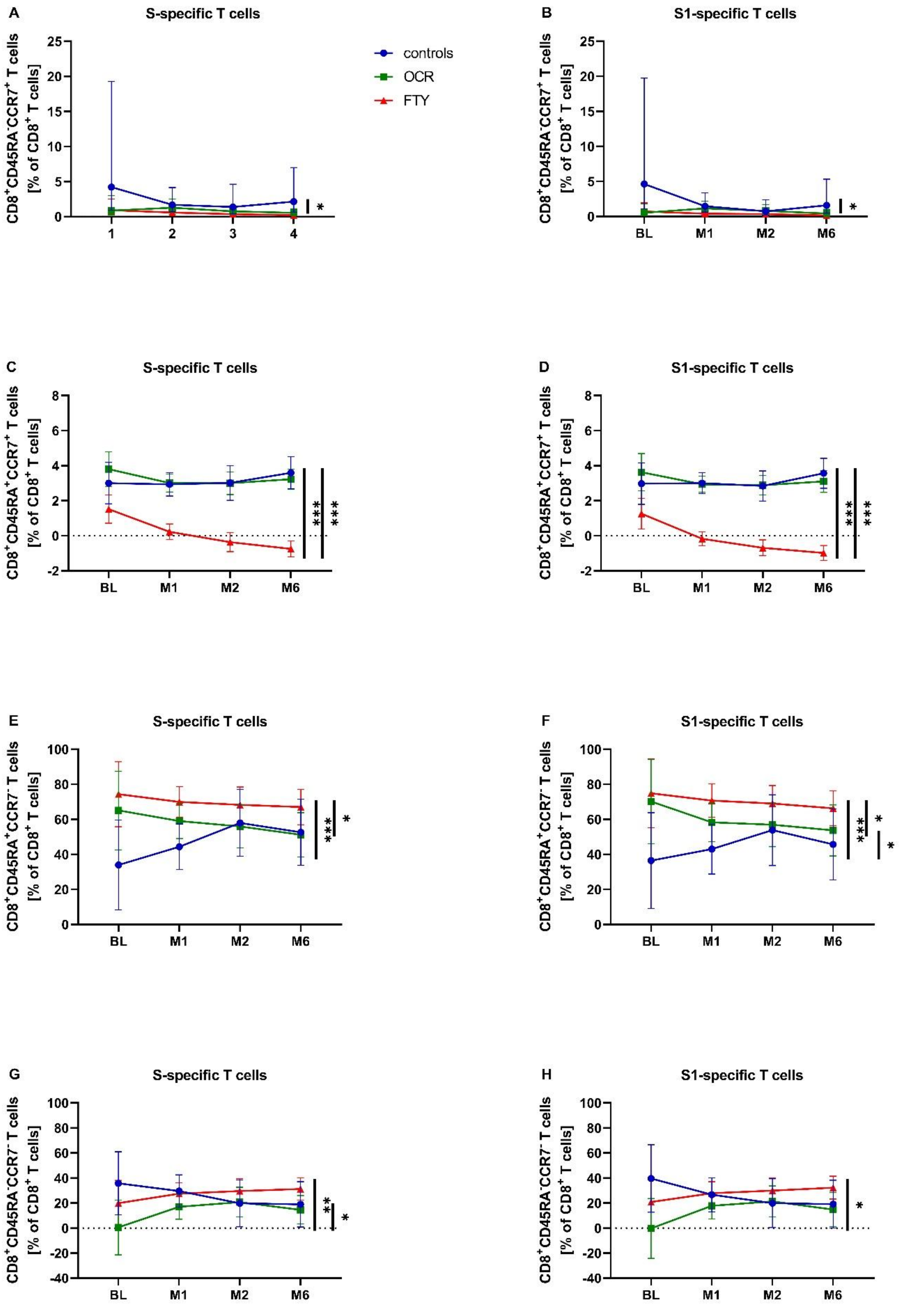

Figure S6. Impact of first SARS-CoV-2 mRNA vaccination on selected spike-specific (S) T-cell subsets and T-cell subsets specific for the S1 domain of the spike protein (S1) in the control group (controls, $n = 17$) compared to the fingolimod (FTY, $n = 15$) and the ocrelizumab (OCR, $n = 15$) group. Relative percentages of T-cell subsets are shown at baseline (BL), one-month follow-up (M1), two-month follow-up (M2) and six-month follow-up (M6). Means with 95% confidence intervals are presented for relative percentages of CD8$^+$CD45RA$^-$CCR7$^+$ T cells **(A,B)**, CD8$^+$CD45RA$^+$CCR7$^+$ T cells **(C,D)**, CD8$^+$CD45RA$^+$CCR7$^-$ cells **(E,F)** and CD8$^+$CD45RA$^-$CCR7$^-$ T cells **(G,H)**. Asterisks indicate a statistically significant difference in percentages of relative T-cell subsets between selected time points or groups (* $p < 0.05$, ** $p < 0.01$, *** $p < 0.001$).

Table S1. Reagents and chemicals.

Reagent/Chemical	Manufacturer (Headquarters)
2-Propanol ≥99,7%, AnalaR NORMAPUR® ACS, Reag. Ph. Eur. analytisches Reagens	VWR International GmbH (Darmstadt, Germany)
BD FACSClean™ Solution	Becton Dickinson GmbH (Heidelberg, Germany)
BD FACSFlow™ Solution	Becton Dickinson GmbH (Heidelberg, Germany)
BD FACSRinse™ Solution	Becton Dickinson GmbH (Heidelberg, Germany)
BD GolgiPlug™ Protein Transport Inhibitor (Containing Brefeldin A)	Becton Dickinson GmbH (Heidelberg, Germany)
BD Pharmingen™ Purified NA/LE Mouse Anti-Human CD28	Becton Dickinson GmbH (Heidelberg, Germany)
BRAUN Aqua ad iniectabilia Wasser für Injektionszwecke	B. Braun SE (Melsungen, Germany)
CytoStim™ human	Miltenyi Biotec B.V. & Co. KG (Bergisch Gladbach, Germany)
Essigsäure (Eisessig) 100%	Merck KGaA (Darmstadt, Germany)
Ethanol 80%, MEK (Butan-2-on)	B. Braun SE (Melsungen, Germany)
Gibco™ FBS (Fetal Bovine Serum) qualified, Brazil	Fisher Scientific GmbH (Schwerte, Germany)
Gibco™ L-Glutamin (200 mM)	Fisher Scientific GmbH (Schwerte, Germany)
Gibco™ Penicillin-Streptomycin	Fisher Scientific GmbH (Schwerte, Germany)
Gibco™ RPMI 1640 Medium	Fisher Scientific GmbH (Schwerte, Germany)
Humanes Serum AB	c.c.pro (Oberdorla, Germany)
Pancoll human, Density: 1.077 g/ml	PAN-Biotech GmbH (Aidenbach, Germany)
Paraformaldehyd	Merck KGaA (Darmstadt, Germany)
PepMix™ HCMVA (pp65)	JPT Peptide Technologies GmbH (Berlin, Germany)
PepTivator® SARS-CoV-2 Prot_M	Miltenyi Biotec B.V. & Co. KG (Bergisch Gladbach, Germany)
PepTivator® SARS-CoV-2 Prot_N	Miltenyi Biotec B.V. & Co. KG (Bergisch Gladbach, Germany)
PepTivator® SARS-CoV-2 Prot_S	Miltenyi Biotec B.V. & Co. KG (Bergisch Gladbach, Germany)
PepTivator® SARS-CoV-2 Prot_S1	Miltenyi Biotec B.V. & Co. KG (Bergisch Gladbach, Germany)
Sigma-Aldrich Dimethylsulphoxid (DMSO) Hybri-Max™	Merck KGaA (Darmstadt, Germany)
Sigma-Aldrich Natriumazid	Merck KGaA (Darmstadt, Germany)
Sigma-Aldrich Saponin Quillaja sp.	Merck KGaA (Darmstadt, Germany)
Supelco 2-Propanolol	Merck KGaA (Darmstadt, Germany)
Trypanblau-Lösung, 0.4%	Merck KGaA (Darmstadt, Germany)
Zombie Green™ Fixable Viability Kit	BioLegend (Koblenz, Germany)

The chemicals and reagents used in present study are shown.

Table S2. Solutions and buffers.

Solution/Buffer	Composition/Manufacturer (Headquarters)
AB Medium without Interleukin(IL)-2	500 mL RPMI 1640<br>25 mL Humanes Serum AB<br>5 mL Penicillin-Streptomycin<br>2,5 mL L-Glutamin (200 mM)<br>Storage: 4°C
BD GolgiPlug™ Protein Transport Inhibitor (Containing Brefeldin A), working solution	10 µL BD GolgiPlug™ Protein Transport Inhibitor<br>990 µL PBS<br>15 µL per well
BD Pharmingen™ Purified NA/LE Mouse Anti-Human CD28, working solution	10 µL Purified NA/LE Mouse Anti-Human CD28<br>650 µL PBS<br>15 µL per well
CytoStim™ human, working solution	100 µL CytoStim™ human<br>2500 µL AB Medium without IL-2<br>10 µL per well
DMSO solution 10%	100 µL DMSO<br>900 µL Aqua dest. sterile<br>Storage: 4 °C
Cryopreservation medium	45 mL FBS inactivated sterile<br>5 mL DMSO 10% sterile<br>2-3 x 10 ^{7} cells per 1 mL of cryopreservation medium<br>Storage: 4 °C
Acetic acid 500 mmol/L for cell counting	7,14 mL acetic acid 100%<br>242,86 mL Aqua ad iniectabilia<br>Storage: in the dark at room temperature (RT)
FACS buffer	500 mL PBS<br>15 mL FBS inactivated<br>5 mL sodium azide 10%<br>Storage: 4°C
Sodium azide solution 10%	10 g sodium azide<br>100 mL Aqua dest.<br>Storage: RT
Paraformaldehyde solution 4%	40 g Paraformaldehyde<br>450 mL Aqua dest.<br>Sodium hydroxide solution 1 mol/L<br>Hydrochloric acid 1 mol/L<br>500 mL PBS<br>2 mL per aliquot<br>Storage: -20°C
PepMix™ HCMVA (pp65), aliquots and working solution	1 vial (25 tests)<br>50 µL DMSO<br>Aliquots of 2 µL each<br>Storage: -20°C;<br>Working solution:<br>Aliquot (2 µL)<br>500 µL AB Medium without IL-2<br>10 µl per well

PepTivator® SARS-CoV-2 Prot_M, aliquots and working solution	60 nmol PepTivator® SARS-CoV-2 Prot_M<br>2 mL 10% DMSO solution<br>Aliquots of 25 μL each<br>Storage: -80°C;<br>Working solution:<br>Aliquot (25 μL)<br>500 μL AB Medium without IL-2<br>10 μL per well
PepTivator® SARS-CoV-2 Prot_N, aliquots and working solution	60 nmol PepTivator® SARS-CoV-2 Prot_N<br>2 mL 10% DMSO solution<br>Aliquots of 25 μL each<br>Storage: - 80°C;<br>Working solution:<br>Aliquot (25 μL)<br>500 μL AB Medium without IL-2<br>Je 10 μL per well
PepTivator® SARS-CoV-2 Prot_S, aliquots and working solution	60 nmol PepTivator® SARS-CoV-2 Prot_S<br>2 mL 10% DMSO solution<br>Aliquots of 25 μL each<br>Storage: -80°C<br>Working solution:<br>Aliquot (25 μL)<br>500 μL AB Medium without IL-2<br>10 μL per well
PepTivator® SARS-CoV-2 Prot_S1, aliquots and working solution	60 nmol PepTivator® SARS-CoV-2 Prot_S1<br>2 mL 10% DMSO solution<br>Aliquots of 25 μL each<br>Storage: -80°C;<br>Working solution:<br>Aliquot (25 μl)<br>500 μL AB Medium without IL-2<br>10 μl per well
Saponin solution 0,1%	500 mL PBS<br>5 mL FBS inactivated<br>5 mL sodium azide 10%<br>5 g Saponin Quillaja sp.<br>Storage: 4 °C
Sigma-Aldrich Dulbeccos Phosphatgepufferte Kochsalzlösung (PBS, modified, without chloride and magnesium chloride)	Merck KGaA (Darmstadt, Germany)<br>Storage: 4 °C
Zombie Green™ Fixable Viability Kit, aliquots and working solution	1 vial (100 tests)<br>100 μL DMSO 10%<br>Aliquots of 25 μL each<br>Storage: -20°C;<br>Working solution:<br>25 μL (Aliquot)<br>990 μL PBS<br>10 μL per well

The solutions and buffers used in present study are shown.

Table S3. Fluorescence-labeled antibodies with quantities.

Fluorescent-labeled antibody (quantity)	Manufacturer (Headquarters)
BD Horizon™ BV421 Mouse Anti-Human TNF (2,5 µL per well)	Becton Dickinson GmbH (Heidelberg, Germany)
BD Horizon™ BV510 Mouse Anti-Human HLA-DR (2,5 µL per well)	Becton Dickinson GmbH (Heidelberg, Germany)
BD Horizon™ BV711 Mouse Anti-Human IL-2 (2,5 µL per well)	Becton Dickinson GmbH (Heidelberg, Germany)
BD Horizon™ BV786 Mouse Anti-Human IFN-γ (2,5 µL per well)	Becton Dickinson GmbH (Heidelberg, Germany)
BD Horizon™ PE-CF594 Mouse Anti-Human CD197 (CCR7) (2,5 µL per well)	Becton Dickinson GmbH (Heidelberg, Germany)
BD Horizon™ PE-CF594 Mouse Anti-Human CD45RA (2,5 µL per well)	Becton Dickinson GmbH (Heidelberg, Germany)
BD Pharmingen™ Alexa Fluor® 700 Mouse Anti-Human CD38 (2,5 µL per well)	Becton Dickinson GmbH (Heidelberg, Germany)
BD Pharmingen™ APC Mouse Anti-Human CD45RA (10 µl per well)	Becton Dickinson GmbH (Heidelberg, Germany)
BD Pharmingen™ APC-H7 Mouse Anti-Human CD3 (2,5 µL per well)	Becton Dickinson GmbH (Heidelberg, Germany)
BD Pharmingen™ PE Mouse Anti-Human CD4 (10 µL per well)	Becton Dickinson GmbH (Heidelberg, Germany)
BD Pharmingen™ PE-Cy 5™ Mouse Anti-Human CD154 (10 µL per well)	Becton Dickinson GmbH (Heidelberg, Germany)
BD Pharmingen™ PerCP-Cy™ 5.5. Mouse Anti-Human CD8 (2,5 µL per well)	Becton Dickinson GmbH (Heidelberg, Germany)

Fluorescent-labeled antibodies along with their quantities used for flow cytometry are shown.

Table S4. Beads with quantities.

Beads	Manufacturer (Headquarters); composition
BD FACSDiva™ CS&T Research Beads	Becton Dickinson GmbH (Heidelberg, Germany);<br>Working solution:<br>1 drop of BD FACSDiva™ CS&T Research Beads<br>360 µL BD FACSFlow™ Solution
BD™ CompBeads Anti-Mouse Ig, κ/Negative Control Compensation Particles Set	Becton Dickinson GmbH (Heidelberg, Germany);<br>Working solution:<br>1 drop of CompBeads Anti-Mouse Ig, κ / 1 drop of Comp Beads Negative Control<br>200 µL FACS-Puffer<br>Respective fluorescent-labeled antibodies in µL according to the quantities specified in Table S3

Beads used for flow cytometry are shown.

Table S5. Group differences in relative percentages of SARS-CoV-2 specific T-cell subsets.

T-cell subsets [%]	Group differences	S	S1
$CD4^+$ (% of $CD3^+$)	Controls to OCR	***	***
	Controls to FTY	***	***
	OCR to FTY	***	***
$CD4^{+high}$ (% of $CD3^+$)	Controls to OCR	*	*
	Controls to FTY	***	***
	OCR to FTY	***	***
$CD4^{+low}$ (% of $CD3^+$)	Controls to OCR	n. s.	n. s.
	Controls to FTY	***	***
	OCR to FTY	***	***
$CD8^+$ (% of $CD3^+$)	Controls to OCR	n. s.	n. s.
	Controls to FTY	***	***
	OCR to FTY	***	***
$CD4^+CD154^+$ (% of $CD4^+$)	Controls to OCR	*	*
	Controls to FTY	n. s.	n. s.
	OCR to FTY	***	***
$CD4^+IFN\text{-}\gamma^+$ (% of $CD4^+$)	Controls to OCR	n. s.	n. s.
	Controls to FTY	n. s.	n. s.
	OCR to FTY	n. s.	n. s.
$CD4^+IL\text{-}2^+$ (% of $CD4^+$)	Controls to OCR	n. s.	n. s.
	Controls to FTY	n. s.	n. s.
	OCR to FTY	n. s.	n. s.
$CD4^+TNF\text{-}\alpha^+$ (% of $CD4^+$)	Controls to OCR	n. s.	n. s.
	Controls to FTY	n. s.	n. s.
	OCR to FTY	n. s.	n. s.

*Significances for group differences between the three patient groups in the percentages of SARS-CoV-2 specific T-cell subsets are shown.

OCR, ocrelizumab, FTY, fingolimod, n. s., not significant

Asterisks indicate a statistically significant difference in relative percentages of T-cell subsets between patient groups (* $p < 0.05$, *** $p < 0.001$).

Table S6. Relative percentages of SARS-CoV-2 specific T-cell subsets in relation to anti-SARS-CoV-2 RBD IgG titers.

T-cell subsets (%)	Patient Group	S		S1	
		IgG +	IgG -	IgG +	IgG -
CD4$^+$CD38$^+$HLA-DR$^+$ (% of CD4$^+$)	All	3.352 CI: (2.144, 5.239)	2.956 CI: (2.047, 4.267)	3.241 CI: (1.934, 5.429)	3.265 CI: (2.328, 4.580)
	Controls	6.483 CI: (3.553, 11.830)	2.098 CI: (0.734, 5.998)	6.369 CI: (3.419, 11.865)	2.765 CI: (1.087, 7.034)
	OCR	2.476** CI: (1.300, 4.719)	5.574** CI: (4.387, 7.081)	2.511** CI: (1.190, 5.301)	5.860** CI: (4.547, 7.552)
	FTY	2.346 CI: (1.516, 3.629)	2.209 CI: (1.681, 2.901)	2.128 CI: (1.294, 3.499)	2.149 CI: (1.616, 2.856)
CD4$^+$CD38$^-$HLA-DR$^-$ (% of CD4$^+$)	All	22.090 CI: (15.295, 31.904)	19.060 CI: (14.498, 25.058)	21.407 CI: (14.581, 31.427)	19.886 CI: (15.049, 26.278)
	Controls	15.133 CI: (3.584, 9.481)	7.604 CI: (3.550, 16.289)	15.970 CI: (9.917, 25.720)	7.592 CI: (3.507, 16.437)
	OCR	9.340 CI: (5.477, 15.925)	13.613 CI: (11.199, 16.547)	7.838* CI: (4.356, 14.106)	14.807* CI: (12.088, 18.138)
	FTY	76.264 CI: (53.218, 109.289)	66.898 CI: (53.470, 83.698)	78.362 CI: (54.196, 113.304)	69.954 CI: (55.591, 88.028)
CD4$^+$CD45RA$^-$CCR7$^+$ (% of CD4$^+$)	All	18.384 CI: (12.234, 24.534)	11.788 CI: (6.493, 17.082)	19.212* CI: (13.038, 25.387)	10.417* CI: (5.552, 15.281)
	Controls	30.271 CI: (22.079, 38.463)	7.739 CI: (-7.380, 22.859)	31.866** CI: (24.063, 39.669)	5.298** CI: (-8.437, 19.033)
	OCR	13.494 CI: (4.986, 22.003)	16.159 CI: (13.087, 19.231)	14.998 CI: (6.157, 23.839)	14.812 CI: (11.791, 17.833)
	FTY	11.386 CI: (5.471, 17.302)	11.464 CI: (7.978, 14.951)	10.773 CI: (4.894, 16.651)	11.140 CI: (7.760, 14.519)
	All	0.557 CI: (0.398, 0.717)	0.766 CI: (0.560, 0.971)	0.826* CI: (0.626, 1.025)	0.515* CI: (0.350, 0.680)
	Controls	0.883	0.428	0.897	0.315

CD8$^+$CD154$^+$ (% of CD8$^+$)		CI: (0.627, 1.140)	CI: (0.003, 0.852)	CI: (0.638, 1.156)	CI: (-0.143, 0.774)
	OCR	0.739 CI: (0.413, 1.066)	0.643 CI: (0.519, 0.767)	0.978 CI: (0.652, 1.304)	0.628 CI: (0.512, 0.745)
	FTY	0.674 CI: (0.472, 0.877)	0.602 CI: (0.456, 0.747)	0.601 CI: (0.410, 0.793)	0.601 CI: (0.466, 0.735)
CD8$^+$IL-2$^+$ (% of CD8$^+$)	All	2.213 CI: (1.607, 2.820)	2.192 CI: (1.644, 2.740)	1.880 CI: (1.366, 2.588)	2.049 CI: (1.650, 2.545)
	Controls	1.826 CI: (0.981, 2.671)	2.250 CI: (0.689, 3.810)	1.664 CI: (1.135, 2.438)	1.948 CI: (1.087, 3.490)
	OCR	3.363* CI: (2.422, 4.303)	2.321* CI: (1.963, 2.678)	3.010 CI: (1.855, 4.886)	2.322 CI: (1.962, 2.750)
	FTY	1.451 CI: (0.860, 2.043)	2.006 CI: (1.594, 2.418)	1.327 CI: (0.976, 1.806)	1.902 CI: (1.569, 2.305)

Impact of first SARS-CoV-2 mRNA vaccination on selected spike (S) specific T-cell subsets and T-cell subsets specific for the S1 domain of the spike protein (S1) as a function of the serostatus of anti-SARS-CoV-2 receptor-binding domain (RBD) immunoglobulin G (IgG) titers. Relative percentages of T-cell subsets are shown for seropositive (IgG +) compared to seronegative (IgG -) individuals. Means with 95% confidence intervals are presented. Mirror-transformed (reflected) data: CD4$^+$CD38$^-$HLA-DR$^-$ T cells

OCR, ocrelizumab; FTY, fingolimod

Asterisks indicate a statistically significant difference in relative percentages of T-cell subsets based on the serostatus in the corresponding patient group (* $p < 0.05$, ** $p < 0.01$).
